# Impact of Tumor Size on Probability of Pathologic Complete Response After Neoadjuvant Chemotherapy

**DOI:** 10.1245/s10434-015-5030-1

**Published:** 2015-12-29

**Authors:** Paul Baron, Peter Beitsch, Danielle Boselli, James Symanowski, James V. Pellicane, Jennifer Beatty, Paul Richards, Angela Mislowsky, Charles Nash, Laura A. Lee, Mary Murray, Femke A. de Snoo, Lisette Stork-Sloots, Mark Gittleman, Stephanie Akbari, Pat Whitworth

**Affiliations:** Department of Surgery, Breast and Melanoma Specialists of Charleston, Charleston, SC USA; Department of Surgery, Dallas Surgical Group, Dallas, TX USA; Department of Biostatistics, Levine Cancer Institute, Charlotte, NC USA; Department of Surgery, Virginia Breast Center, Bon Secours Cancer Institute, Richmond, VA USA; Department of Surgery, The Breast Place, Charleston, SC USA; Department of Surgery, Blue Ridge Cancer Care, Roanoke, VA USA; Department of Surgery, Coastal Carolina Breast Center, Murrells Inlet, SC USA; Department of Surgery, Northeast Georgia Medical Center, Gainesville, GA USA; Department of Surgery, Comprehensive Cancer Center, Palm Springs, CA USA; Department of Surgery, Akron General Hospital, Akron, OH USA; Department of Medical Affairs, Agendia Inc, Irvine, CA USA; Department of Surgery, Breast Care Specialists, Allentown, PA USA; Department of Surgery, Virginia Hospital Center, Arlington, VA USA; Department of Surgery, Nashville Breast Center, Nashville, TN USA

## Abstract

**Background:**

The prospective Neoadjuvant Breast Symphony Trial (NBRST) study found that MammaPrint/BluePrint functional molecular subtype is superior to conventional immunohistochemistry/fluorescence in situ hybridization subtyping for predicting pathologic complete response (pCR) to neoadjuvant chemotherapy. The purpose of this substudy was to determine if the rate of pCR is affected by tumor size.

**Methods:**

The NBRST study includes breast cancer patients who received neoadjuvant chemotherapy. MammaPrint/BluePrint subtyping classified patients into four molecular subgroups: Luminal A, Luminal B, HER2 (human epidermal growth factor receptor 2), and Basal type. Probability of pCR (ypT0/isN0) as a function of tumor size and molecular subgroup was evaluated.

**Results:**

A total of 608 patients were evaluable with overall pCR rates of 28.5 %. Luminal A and B patients had significantly lower rates of pCR (6.1 and 8.7 %, respectively) than either basal (37.1 %) or HER2 (55.0 %) patients (*p* < 0.001). The probability of pCR significantly decreased with tumor size >5 cm [*p* = 0.022, odds ratio (OR) 0.58, 95 % confidence interval (CI) 0.36, 0.93]. This relationship was statistically significant in the Basal (*p* = 0.026, OR 0.46, 95 % CI 0.23, 0.91) and HER2 (*p* = 0.039, OR 0.36, 95 % CI 0.14, 0.95) subgroups. In multivariate logistic regression analyses, the dichotomized tumor size variable was not significant in any of the molecular subgroups.

**Discussion:**

Even though tumor size would intuitively be a clinical determinant of pCR, the current analysis showed that the adjusted OR for tumor size was not statistically significant in any of the molecular subgroups. Factors significantly associated with pCR were PR status, grade, lymph node status, and BluePrint molecular subtyping, which had the strongest correlation.

Neoadjuvant chemotherapy (NCT) was initially shown to downsize many large or locally advanced breast cancers, thus increasing the likelihood of clear margins with a mastectomy or lumpectomy. For triple-negative and HER2 (human epidermal growth factor receptor 2) tumors, pathologic complete response (pCR) correlates with improved survival.^1^–^5^

The Neoadjuvant BReast Symphony Trial (NBRST) found that MammaPrint/BluePrint molecular subtyping reclassifies 22 % (94 of 426) of tumors. MammaPrint/BluePrint molecular subtyping reassigned patients with more responsive disease to the HER2 and Basal categories while reassigning patients with less responsive disease to the Luminal categories. These findings suggest that compared to immunohistochemistry (IHC)/fluorescence in situ hybridization (FISH), MammaPrint/BluePrint more accurately identifies patients with disease likely to respond or not respond to NCT.^6^

NCT is increasingly being adopted in the clinical management of patients with more locally advanced disease and/or more aggressive tumor types. Several identified reasons for this trend include the following: an opportunity to observe tumor response to chemotherapy; improvement in operability; and association of improved survival for more aggressive subtypes in patients who experience pCR.^1^–^5^ In light of improvements in NCT regimens, there has been an increased use of this approach in patients with smaller tumors, who in the past would have more commonly gone straight to surgical lumpectomy. This has generally been seen in patients with either triple-negative or HER2 tumors with the hope that it would lead to a pCR and presumably better long-term survival.

Intuitively, it seems that patients with smaller tumors would more often experience a pCR than those with larger tumors, which would in turn lead to an additional survival benefit. Thus, the purpose of this unplanned substudy was to determine if the pCR rate is also affected by tumor size and if the tumor size effect is modified by molecular subtype as determined by BluePrint molecular subtyping.

## Patients and Methods

### Patients

Patients with histologically proven breast cancer, who had started or were scheduled to start NCT therapy or neoadjuvant hormone therapy, after successful MammaPrint/BluePrint assay were enrolled onto the prospective NBRST registry trial between June 2011 and November 2014 from 62 U.S. institutions. The trial was approved by institutional review boards in all participating centers and registered with ClinicalTrials.gov (NCT01479101). Before registration, all patients provided signed informed consent for the trial and for research on their tumor samples. Excluded from the study were patients who had an excisional biopsy or axillary dissection; patients with confirmed distant metastatic disease; patients with any prior chemotherapy, radiotherapy, or endocrine therapy for the treatment of breast cancer; and any serious uncontrolled intercurrent infections or other serious uncontrolled comorbid disease. Treatment was at the discretion of the physician adhering to either National Comprehensive Cancer Network—approved or other peer-reviewed, established regimens. No specific recommendations were given for the selection to treat patients with neoadjuvant treatment. The NBRST registry is a unique, large, real-world database of U.S. patients treated in high-volume breast programs that provides insight into physician choices for this neoadjuvant treatment—eligible population. For the current substudy, patients treated with neoadjuvant endocrine therapy were excluded; only patients with invasive ductal carcinoma were included.

### Molecular and Clinical Characteristics

The 70-gene expression profile MammaPrint and the 80-gene molecular subtyping profile BluePrint were assessed from the fresh or formalin-fixed, paraffin-embedded core needle biopsy at the centralized Agendia Laboratory blinded for clinical and pathologic data. Microarray analysis (RNA labeling, microarray hybridization, and scanning) was performed on the RNA, which was cohybridized with a standard reference to the custom-designed diagnostic chip, each containing oligonucleotide probes for the profiles in triplicate or more.^7^,^8^

Four distinct molecular subgroups—Luminal A, Luminal B, HER2, and Basal—were identified and used for further analysis. In this study, we defined Luminal A-type tumors as Luminal type by BluePrint with a Low Risk score by MammaPrint, and Luminal B-type tumors as BluePrint Luminal type with a MammaPrint High Risk score.

Hormone receptor status [estrogen receptor (ER) and progesterone receptor (PR) status] and HER2 status were determined locally on pretreatment core biopsy samples. Both ER and PR status were determined by IHC and were considered positive if there was ≥1 % positive staining. HER2 status was determined by IHC and/or FISH assays locally. HER2 status was regarded as positive if there was 3+ staining and/or FISH positivity according to American Society of Clinical Oncology/College of American Pathologists HER2 testing guidelines at the time of diagnosis.

### Objectives and Endpoints

The primary study endpoint was pCR, defined as the absence of invasive carcinoma in both the breast and axilla at microscopic examination of the resection specimen, regardless of the presence of carcinoma-in situ (ypT0/isN0). All patients underwent pretreatment imaging of their primary tumor performed. The largest pre-NCT tumor size measurement from diagnostically used mammography, ultrasound, magnetic resonance imaging, positron emission tomography combined with computed tomography, positron emission mammography, or computed tomography was used. T stage was determined by the treating physician according to the American Joint Committee on Cancer (AJCC) 7th edition breast cancer staging.^6^

To determine whether lymph node status was relevant in the current analysis, we also analyzed the data using a definition of pCR in which lymph node status was not included (ypTis/0).

### Statistical Analysis

Rates of pCR were calculated for each MammaPrint/BluePrint molecular subtype, tumor size subgroup, and tumor size by molecular subtype; the pCR rates are presented as proportions of the indicated subgroup.

Logistic regression was used to model the probability of pCR as a function of tumor size. This was modeled for the entire cohort. The odds ratio (OR) estimated from the logistic regression analysis was associated with a 2.1 cm change in tumor size (approximately equal to 1 standard deviation). In an effort to make an easily interpretable and useful tool for clinicians, we sought to establish a dichotomous variable for tumor size in order to evaluate differential pCR rates with respect to tumor size in specific molecular subtypes. Regarding the AJCC staging system, description of the primary tumor involves both size and extent of the tumor; the largest tumor diameter of T1 and T2 staged tumors is necessarily less than or equal to 5 cm. Logistic regression was therefore used to model the probability of pCR as a function of a tumor size variable dichotomized at 5 cm in the entire cohort and in each molecular subtype separately. Univariate logistic regression analyses of pCR were evaluated to identify individual patient and tumor prognostic factors. Significant factors from the univariate analyses were included in a multivariate modeling procedure in the overall cohort. Backward elimination followed by forward selection was performed to identify independently prognostic factors. The dichotomized tumor size variable was then included in the multivariate model to estimate an adjusted tumor size ORs for each of the three BluePrint molecular subtypes.

Fisher’s exact test was used to compare pCR rates by molecular subgroup and by T stage.

All calculations were performed by SAS 9.3 (SAS Institute, Cary, NC).

## Results

Table 1 lists the pretreatment patient and tumor characteristics for the 608 evaluable patients per MammaPrint/BluePrint Molecular Subtyping group. The median age of the patients was 52 years.

**Table 1:** Baseline patient characteristics by MammaPrint/BluePrint molecular subtyping group

Characteristic	All patients (*n* = 608)	Patients by subtype			
		Luminal A (*n* = 66)	Luminal B (*n* = 183)	HER2 (*n* = 111)	Basal (*n* = 248)
Median age (range)	52 (18–89)	51 (33–69)	54 (22–79)	49 (23–81)	52 (18–89)
Clinical tumor size (mm)					
Median (range)	33 (7–122)	32 (7–110)	35 (10–120)	31 (9–100)	31 (7–122)
Tumor stage					
cT1	78 (13 %)	4 (6 %)	22 (12 %)	14 (13 %)	38 (15 %)
cT2	374 (62 %)	42 (64 %)	116 (63 %)	65 (59 %)	151 (61 %)
cT3	133 (22 %)	17 (26 %)	37 (20 %)	26 (23 %)	53 (21 %)
cT4	23 (4 %)	3 (5 %)	8 (4 %)	6 (5 %)	6 (2 %)
Nodal stage					
cN0	228 (38 %)	27 (41 %)	48 (26 %)	38 (34 %)	115 (46 %)
cN1	308 (51 %)	33 (50 %)	109 (60 %)	62 (56 %)	104 (42 %)
cN2	31 (5 %)	2 (3 %)	12 (7 %)	4 (4 %)	13 (5 %)
cN3	18 (3 %)	2 (3 %)	3 (2 %)	2 (2 %)	11 (4 %)
Missing	23 (4 %)	2 (3 %)	11 (6 %)	5 (5 %)	5 (2 %)
Tumor grade					
1	25 (4 %)	12 (18 %)	10 (5 %)	2 (2 %)	1 (< 1 %)
2	188 (31 %)	41 (62 %)	73 (40 %)	40 (36 %)	34 (14 %)
3	384 (63 %)	12 (18 %)	94 (51 %)	66 (59 %)	212 (85 %)
Missing	11 (2 %)	1 (2 %)	6 (3 %)	3 (3 %)	1 (< 1 %)
ER status (IHC)					
Negative	242 (40 %)	1 (2 %)	2 (1 %)	55 (50 %)	184 (74 %)
Positive	364 (60 %)	65 (98 %)	181 (99 %)	56 (50 %)	62 (25 %)
Missing	2 (<1 %)	0	0	0	2 (1 %)
PR status (IHC)					
Negative	316 (52 %)	0	25 (14 %)	75 (68 %)	216 (87 %)
Positive	290 (48 %)	66 (100 %)	158 (86 %)	36 (32 %)	30 (12 %)
Missing	2 (<1 %)	0	0	0	2 (1 %)
HER2 status (IHC/FISH)					
Negative	404 (66 %)	54 (82 %)	132 (72 %)	1 (1 %)	217 (88 %)
Positive	201 (33 %)	12 (18 %)	51 (28 %)	110 (99 %)	28 (11 %)
Missing	3 (< 1 %)	0	0	0	3 (1 %)
MammaPrint					
Low Risk	68 (11 %)	66 (100 %)	0	2 (2 %)	0
High Risk	540 (89 %)	0	183 (100 %)	109 (98 %)	248 (100 %)
Neoadjuvant treatment					
AC > T	259 (43 %)	39 (59 %)	94 (51 %)	1 (1 %)	125 (50 %)
TC	59 (10 %)	9 (14 %)	24 (13 %)	0 (0 %)	26 (10 %)
TAC	48 (8 %)	4 (6 %)	13 (7 %)	2 (2 %)	27 (11 %)
TCH	95 (16 %)	2 (3 %)	25 (14 %)	53 (48 %)	15 (6 %)
AC > TH	43 (7 %)	5 (8 %)	8 (4 %)	55 (50 %)	8 (3 %)
THCP	34 (6 %)	4 (7 %)	10 (5 %)	17 (15 %)	3 (1 %)
Other	73 (12 %)	6 (9 %)	9 (5 %)	16 (14 %)	44 (18 %)
Surgery					
Mastectomy	346 (57 %)	40 (61 %)	102 (56 %)	65 (59 %)	139 (56 %)
Lumpectomy	262 (43 %)	26 (39 %)	81 (44 %)	46 (41 %)	109 (44 %)
Imaging (before NCT)					
MRI	287 (47 %)	27 (41 %)	95 (52 %)	51 (46 %)	114 (46 %)
Mammogram	151 (25 %)	23 (35 %)	47 (26 %)	32 (29 %)	49 (20 %)
Ultrasound	135 (22 %)	16 (24 %)	33 (18 %)	22 (20 %)	64 (26 %)
PET	18 (3 %)	0	6 (3 %)	2 (2 %)	10 (4 %)
Other	17 (3 %)	0	2 (1 %)	4 (4 %)	11 (4 %)

*ER* estrogen receptor, *PR* progesterone receptor, *HER2* human epidermal growth factor receptor 2, *IHC* immunohistochemistry, *FISH* fluorescence in situ hybridization, *A* doxorubicin, *T* taxane, *C* cyclophosphamide, *H* trastuzumab, *P* pertuzumab, *THCP* docetaxel–carboplatin–trastuzumab–pertuzumab, *NCT* neoadjuvant chemotherapy, *MRI* magnetic resonance imaging, *PET* positron emission tomography

Tumor ER and PR status at diagnosis was determined in tumor samples from 606 patients; 60.1 % were ER positive and 47.9 % were PR positive. HER2 status at diagnosis was determined in tumor samples from 605 patients, and 33.2 % were HER2 positive. The median largest diameter of the primary tumor was 33 mm, ranging from 7 to 122 mm. A total of 25.7 % of patients presented with T3 or T4 tumors, and 61.0 % of the patients presented with clinically node-positive disease. Data analysis revealed that 95.8 % had tumors of intermediate or high histologic grade.

Review of the chemotherapy regimens showed that the most commonly used regimens in clinical HER2-negative patients were: 59 % AC (doxorubicin/cyclophosphamide) followed by a taxane, 15 % TC (docetaxel/cyclophosphamide), and 11 % TAC (docetaxel/doxorubicin/cyclophosphamide). Of the HER2-enriched patients, 96 % received trastuzumab simultaneously with NCT, and 26 % of these patients received trastuzumab and pertuzumab. Before U.S. Food and Drug Administration approval of pertuzumab in the neoadjuvant setting (September 2013), 57 % received TCH (docetaxel/carboplatin/trastuzumab), and 28 % received AC followed by TH (doxorubicin/cyclophosphamide followed by docetaxel/trastuzumab). After September 2013, the following regimen were mostly used: 50 % TCHP (docetaxel/carboplatin/trastuzumab/pertuzumab), 20 % TCH (docetaxel/carboplatin/trastuzumab), and 10 % THP (docetaxel/trastuzumab/pertuzumab).

A total of 89 % of patients completed their planned NCT without any modifications. Eight percent had dose modifications because of toxicities, 2 % stopped NCT early because of tumor progression, and no reason was specified for the remaining 1 %.

The overall pCR (ypT0/isN0) rate was 28.5 %. Comparison of pCR rates across the four MammaPrint/BluePrint molecular subgroups (on 3 degrees of freedom) was highly significant (*p* < 0.001). Luminal A tumors had 6.1 % pCR rate, which was not statistically significantly different (*p* = 0.604) from the pCR rate of 8.7 % in Luminal B tumors. The pCR rates for Luminal A and B subtypes were statistically significantly less than the pCR rates for Basal (*p* < 0.001 and *p* < 0.001, respectively) and HER2 subtypes (*p* < 0.001 and *p* < 0.001, respectively). The pCR rates for Basal (37.1 %) and HER2 (55.0 %) tumors also differed significantly (*p* = 0.002).

**Fig. 1:**
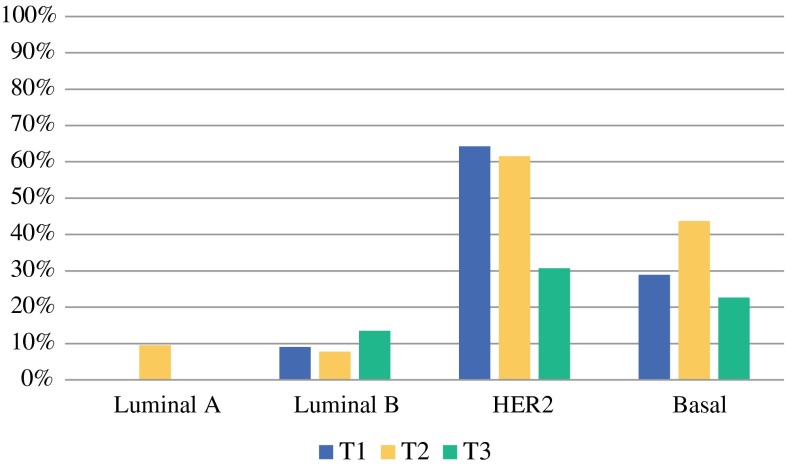
pCR (ypT0/isN0) rate according by clinical T stage and MammaPrint/BluePrint molecular subtyping group (excluding T4)

The rates for experiencing pCR by clinical T stage were as follows: T1 28.2 % (22 of 78), T2 31.8 % (119 of 374), T3 18.8 % (25 of 133), and T4 30.4 % (7 of 23) (Fisher’s exact test, *p* = 0.035). Moreover, the pCR rate for T1 and T2 stage tumors combined (31.2 %) was statistically significantly higher (*p* = 0.006) than the observed pCR rate in T3 stage tumors (18.8 %). Figure 1 shows the pCR rate according by clinical T stage and molecular subtype (excluding T4).

**Fig. 2:**
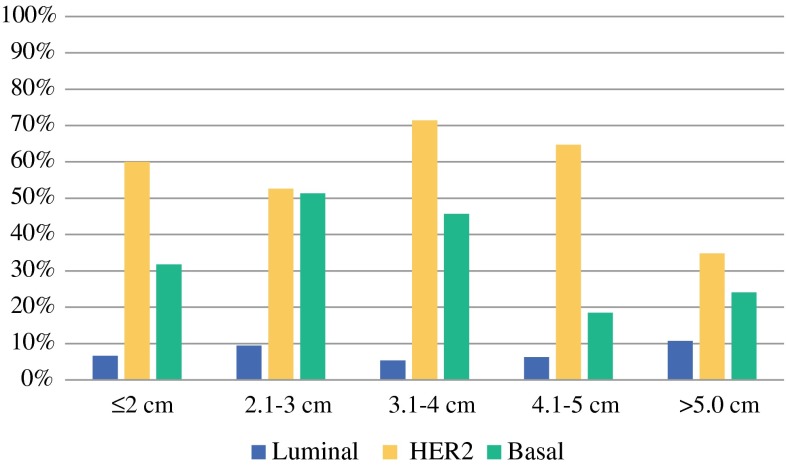
pCR (ypT0/isN0) rate according to tumor size and BluePrint subtype

There were 602 patients with known tumor size included in the tumor size analyses. Because the pCR rate failed to be statistically significantly different and was low among the Luminal A and B subgroups, we decided to pool Luminal A and B in the analysis of tumor size. Subsequent analyses of tumor size were across three BluePrint molecular subgroups: pooled Luminal, HER2, and Basal. The probability of pCR significantly decreased with increasing tumor size as a continuous measure (*p* = 0.027). The OR of pCR associated with a 2.1 cm difference in tumor size (approximately 1 standard deviation) was 0.80 [95 % confidence interval (CI) 0.66, 0.98] (Fig. 2; Table 2). Analysis of the tumor size variable dichotomized at ≤5 versus >5 cm indicated the probability of pCR was significantly decreased in tumors >5 cm relative to smaller tumors (*p* = 0.022, OR 0.58, 95 % CI 0.36, 0.93).

**Table 2:** pCR (ypT0/isN0) rate by tumor size and MammaPrint/BluePrint molecular subtyping group

Tumor size	No. pCR/total (%)	No. pCR/total (%) per MammaPrint/BluePrint subtyping group			
		Luminal A	Luminal B	HER2	Basal
≤2 cm	25/89 (28 %)	0/8 (0 %)	2/22 (9 %)	9/15 (60 %)	14/44 (32 %)
2.1–3 cm	66/188 (35 %)	4/22 (18 %)	3/52 (6 %)	20/38 (53 %)	39/76 (51 %)
3.1–4 cm	34/116 (29 %)	0/14 (0 %)	3/42 (7 %)	10/14 (71 %)	21/46 (46 %)
4.1–5 cm	18/76 (24 %)	0/7 (0 %)	2/25 (8 %)	11/17 (65 %)	5/27 (19 %)
>5.0 cm	27/133 (20 %)	0/15 (0 %)	6/41 (15 %)	8/23 (35 %)	13/54 (24 %)
Total	170/602 (28 %)	4/66 (6 %)	16/182 (9 %)	58/107 (54 %)	92/247 (37 %)
Odds ratio for pCR^a^ (95 % CI)	0.58 (0.36, 0.93)	1.53 (0.56, 4.17)		0.36 (0.14, 0.95)	0.46 (0.23, 0.91)

*pCR* pathologic complete response, *CI* confidence interval

^a^Odds ratio for pCR associated with tumors with size >5 cm relative to ≤5 cm

With significant difference in overall pCR rate for tumor size >5 versus ≤5 cm, we investigated the relationship of tumor size dichotomized at 5 cm with probability of experiencing pCR in subset analyses of molecular subgroups. When analyzed by BluePrint molecular subgroup, this relationship was statistically significant in the Basal subgroup (*p* = 0.026, OR 0.46, 95 % CI 0.23, 0.91) and the HER2 subgroup (*p* = 0.039, OR 0.36, 95 % CI 0.14, 0.95). In comparison, the dichotomized tumor size variable did not correlate with pCR rate in the pooled Luminal subgroup (*p* = 0.411).

The following factors were found to be significantly (*p* < 0.05) associated with the odds of experiencing pCR based on univariate logistic regression analyses (Table 3): clinical lymph node status, clinical tumor stage, tumor grade, ER status, PR status, HER2 status, MammaPrint result, and BluePrint result. The following factors were independently associated with the odds of experiencing pCR based on multivariate logistic regression modeling: clinical lymph node status, tumor grade, PR status, HER2 status, and BluePrint result. When the dichotomized tumor size variable was added to the multivariate base model and assessed within each BluePrint molecular subtype, the adjusted ORs for tumor size >5 versus ≤5 cm tumor were not significant in any of the BluePrint molecular subgroups (Basal subgroup, OR 0.56, 95 % CI 0.27, 1.17, *p* = 0.123; HER2 subgroup, OR 0.44, 95 % CI 0.15, 1.24, *p* = 0.119; Luminal subgroup, OR 1.9, 95 % CI 0.59, 6.17, *p* = 0.286).

**Table 3:** Univariate analysis of patient and tumor characteristics associated with pCR (ypT0/isN0) versus incomplete pathologic primary tumor and axillary lymph node response to NCT

Characteristic	pCR	Incomplete response	Univariate *p* value	Multivariate *p* value	Multivariate OR (95 % CI)
All patients	173 (28 %)	435 (72 %)			
Patient age					
≤50 years	81 (29 %)	199 (71 %)	0.811		
>50 years	92 (28 %)	236 (72 %)			
IHC ER status at diagnosis^a^					
Positive	65 (18 %)	299 (82 %)	<0.001		
Negative	107 (44 %)	135 (56 %)			
PR status at diagnosis^a^					
Positive	38 (13 %)	252 (87 %)	<0.001	0.025	0.51 (0.28, 0.92)
Negative	134 (42 %)	182 (58 %)			Ref.
HER2 status at diagnosis^a^					
Positive	84 (42 %)	117 (58 %)	<0.001	<0.001	2.70 (1.72, 4.21)
Negative	88 (22 %)	316 (78 %)			Ref.
Grade at diagnosis^a^					
1	1 (4 %)	24 (96 %)	<0.001	0.025	0.22 (0.03, 1.80)
2	34 (18 %)	154 (82 %)			0.52 (0.30, 0.88)
3	134 (35 %)	250 (65 %)			Ref.
T stage					
T1	22 (28 %)	56 (72 %)	0.046		
T2	119 (32 %)	255 (68 %)			
T3	25 (19 %)	108 (81 %)			
T4	7 (30 %)	16 (70 %)			
Initial lymph node status^a^					
Negative	90 (39 %)	138 (61 %)	<0.001	<0.001	2.08 (1.36, 3.21)
Positive	78 (22 %)	279 (78 %)			Ref.
BluePrint-subtype status					
Non-Luminal	153 (43 %)	206 (57 %)	<0.001	<0.001	4.21 (2.10, 8.42)
Luminal	20 (8 %)	229 (92 %)			Ref.
MammaPrint					
Low Risk	5 (7 %)	63 (93 %)	<0.001		
High Risk	168 (31 %)	372 (69 %)			

*pCR* pathologic complete response, *NCT* neoadjuvant chemotherapy, *OR* odds ratio, *CI* confidence interval, *IHC* immunohistochemistry, *PR* progesterone receptor, *ER* estrogen receptor

^a^Included in multivariate modeling of dichotomized tumor size and molecular subtype

With the use of the pCR definition excluding nodal status (ypT0/is), the pCR rates were slightly higher; however, there was no difference in statistically significant versus nonsignificant analyses (data not shown).

## Discussion

The NBRST registry provides a unique opportunity to study whether smaller tumors are likely to demonstrate pCR. This would imply an associated improved survival. In this current analysis of the NBRST study, we found that although size was correlated inversely with the frequency of pCR in BluePrint HER2 and Basal subtypes, this was not demonstrated in the multivariate logistic regression analyses, where BluePrint molecular subtype was the strongest of the characteristics associated with pCR (grade, HER2, PR, and lymph node status).

Patients who experience pCR defined as ypT0 ypN0 or ypT0/is ypN0 have improved survival.^1^,^3^ In our prospective registry study, we used the recommended definition of pCR (ypT0/isN0). All pCRs were verified with a deidentified copy of the surgical pathology report. The overall pCR rate to NCT of 28 % in our study is higher than the pCR rate of 20 % using the same definition in recently published meta-analyses.^1^,^3^ This may be because in our study almost all clinical HER2-positive patients received trastuzumab, while this was not yet the case in the pooled meta-analyses. In the meta-analyses of Cortazar et al., patients with clinical T1 or T2 tumors had nonstatistically significant higher pCR rates than patients with T3 and T4 tumors. Furthermore, clinical tumor size was not significantly correlated with an increase in overall survival.^3^ Another study investigating clinical characteristics and pCR association found an association of tumor size and pCR in univariate analysis, which disappeared in multivariate analysis.^9^

Tumor size and lymph node status are the two most important determinants in the AJCC staging manual. Tumor size correlates with long-term survival, as patients with smaller tumors have a lower T stage and generally a better long-term prognosis from their breast cancer compared to those with larger tumors and a higher T stage.^10^

Whitworth et al. have previously reported in the NBRST study on the impact of MammaPrint/BluePrint molecular subtypes and the frequency of pCR in contrast to clinical subtypes (derived by ER, PR, and HER2). Luminal A and B tumors do not usually respond with a pCR to NCT, while pCR rates are significantly higher in HER2 and Basal subtypes.^6^ Some ER-positive highly proliferative tumors do respond to chemotherapy; indeed, identification of patients who are best treated by endocrine therapy versus cytotoxic chemotherapy is an area of great current interest and active investigation in clinical trials.

It also has been shown that patients with HER2-positive or triple-negative cancers are more likely to experience improved long-term survival if they have a pCR after NCT.^1^–^5^

Even though tumor size would intuitively be a clinical determinant of pCR, the current unplanned subanalysis in the prospective neoadjuvant NBRST study showed that the adjusted OR for tumor size was not statistically significant in any of the BluePrint molecular subgroups. Factors significantly associated with pCR were PR status, HER2 status, grade, lymph node status, and BluePrint molecular subtyping, which had the strongest correlation.
